# *Escherichia albertii*, a novel human enteropathogen, colonizes rat enterocytes and translocates to extra-intestinal sites

**DOI:** 10.1371/journal.pone.0171385

**Published:** 2017-02-08

**Authors:** Denise Yamamoto, Rodrigo T. Hernandes, Ana Maria A. Liberatore, Cecilia M. Abe, Rodrigo B. de Souza, Fabiano T. Romão, Vanessa Sperandio, Ivan H. Koh, Tânia A. T. Gomes

**Affiliations:** 1 Departamento de Microbiologia, Imunologia e Parasitologia, Universidade Federal de São Paulo, Escola Paulista de Medicina (UNIFESP-EPM), São Paulo, Brazil; 2 Departamento de Microbiologia e Imunologia, Instituto de Biociência, Universidade Estadual Paulista Júlio de Mesquita Filho (UNESP), Botucatu, São Paulo, Brazil; 3 Departamento de Cirurgia, Universidade Federal de São Paulo, Escola Paulista de Medicina (UNIFESP-EPM), São Paulo, Brazil; 4 Laboratório de Biologia Celular, Instituto Butantan, São Paulo, Brazil; 5 Department of Microbiology and Biochemistry, University of Texas Southwestern Medical Center, Dallas, Texas, United States of America; Universitat Osnabruck, GERMANY

## Abstract

Diarrhea is the second leading cause of death of children up to five years old in the developing countries. Among the etiological diarrheal agents are atypical enteropathogenic *Escherichia coli* (aEPEC), one of the diarrheagenic *E*. *coli* pathotypes that affects children and adults, even in developed countries. Currently, genotypic and biochemical approaches have helped to demonstrate that some strains classified as aEPEC are actually *E*. *albertii*, a recently recognized human enteropathogen. Studies on particular strains are necessary to explore their virulence potential in order to further understand the underlying mechanisms of *E*. *albertii* infections. Here we demonstrated for the first time that infection of fragments of rat intestinal mucosa is a useful tool to study the initial steps of *E*. *albertii* colonization. We also observed that an *E*. *albertii* strain can translocate from the intestinal lumen to Mesenteric Lymph Nodes and liver in a rat model. Based on our finding of bacterial translocation, we investigated how *E*. *albertii* might cross the intestinal epithelium by performing infections of M-like cells *in vitro* to identify the potential *in vivo* translocation route. Altogether, our approaches allowed us to draft a general *E*. *albertii* infection route from the colonization till the bacterial spreading *in vivo*.

## Introduction

*Escherichia albertii* is a recently recognized close relative of *Escherichia coli*, which is an emerging human enteropathogen and avian pathogen [1,2]. Since it is difficult to discriminate from other species of *Enterobacteriaceae* by common biochemical identification assays [3], *E*. *albertii* isolates can be erroneously identified as *Hafnia alvei*, *Shigella boydii*, *Yersinia ruckeri* or *E*. *coli* [4–6].

Diarrheagenic *E*. *coli* (DEC) are classified into different categories according to their set of virulence genes. Enteropathogenic *E*. *coli* (EPEC), one of the DEC categories, are sub-grouped into typical (tEPEC) and atypical (aEPEC) based on the presence of the *bfp* operon (encoding the bundle forming pilus—BFP) in tEPEC [7,8] and its absence in aEPEC ([9] and reviewed in [10]).

Both EPEC groups use the type 3 secretion system (T3SS) to inject a number of effector proteins directly into the host cell, including Tir (translocated intimin receptor), which is inserted into the host cell membrane and acts as a receptor for the EPEC adhesin called intimin [11–13]. Altogether, these events promote microvilli effacement and intimate bacterial adherence to the enterocyte membrane leading to the so-called attaching and effacing (AE) lesion, in which pedestal-like structures that are rich in actin and other cytoskeleton components are formed [14–16]. The T3SS, Tir and Intimin-encoding genes are located in a pathogenicity island named the locus of enterocyte-effacement (LEE). Besides the Tir-intimin interaction, the presence of other potential adhesins such as *E*. *coli* common pilus (ECP) and type 1 pilus (T1P) have been reported in aEPEC strains [17–19].

We have previously shown that certain aEPEC strains may invade cultured cells *in vitro* [20–24] and that the aEPEC 1551–2 strain invades enterocytes through the basolateral surface more effectively than through the apical surface [22]. Invasive enteropathogens can reach the basolateral receptors and promote host cell invasion *in vivo* by transcytosis through M cells [25]. M cells are recognized as part of the innate immune response (reviewed by [26,27]), and are found on Peyer’s patches (gut associated lymphoid tissue—GALT) in association with phagocytic cells, forming the follicle-associated epithelium (FAE) [26,27]. Hase et al., [28] demonstrated that deficiency of bacterial FimH (the T1P adhesin) or its receptor, host glycoprotein 2 (GP2), led to defects in bacterial transcytosis through M cells, resulting in an attenuation of antigen-specific immune responses in Peyer’s patches.

There is little clinical evidence, but experimental data have indicated that, at least in the initial infection steps, M cells transport pathogens from the intestinal lumen to macrophages in the lamina propria of the mucosa [29]. In fact, some enteropathogens such as *Shigella*, *Salmonella* and *Yersinia* use M cells as the main entrance site in enterocytes [29,30]. After the translocation through M cells, *Shigella* is phagocytized, leading to the apoptosis of macrophages, and is released to access the enterocyte basolateral surface [31].

During a recent evaluation of a collection of aEPEC strains in our laboratory, using a polymerase chain reaction system [32] and a preliminary analysis of the genomic sequence of the LEE region of one strain (1551–2 strain), we have found out that six aEPEC strains, including the 1551–2 strain, are actually *E*. *albertii* ([6] and unpublished data).

It is known that *E*. *albertii* strains share with EPEC and EHEC (Enterohemorrhagic *E*. *coli*) the ability to promote AE lesions due to the presence of the LEE [4,6]. However, there is scarce information regarding the virulence mechanisms that favor the interaction of *E*. *albertii* with the intestinal mucosa [1].

Host colonization of the small and/or large intestines comprises the first step in the establishment of diarrheal diseases by bacterial pathogens, such as *Vibrio cholera*, EPEC and *Salmonella* spp. [30]. However, studies on the interaction of *E*. *albertii* strains of human origin with enterocytes are scarce and appropriate animal models to explore the interaction of *E*. *albertii* strains with the intestinal mucosa have not been described [33,34]. To contribute to this issue, we evaluated the rat intestinal mucosa as a model to study *E*. *albertii* adhesion, colonization and translocation from the intestinal lumen to extra-intestinal sites. In addition, we investigated the participation of the intimin-Tir interaction, the T3SS-translocon and T1P in this infection model, as well as *E*. *albertii* translocation into M-like cells.

## Materials and methods

### Ethics statement

The protocols involving animal handling were approved by the Research Ethics Committee of UNIFESP, project license number 0342/09. “Comitê de Ética em Pesquisa da UNIFESP/ Hospital São Paulo” (CEP UNIFESP/HU-HSP) is in accordance with Good Clinical Practice (GCP) of the International Council for Harmonisation (ICH), formerly the International Conference on Harmonisation (ICH). Animals are handled under "Brazilian Guidelines For The Care And Use Of Animals In Educational Activities Or Scientific Research" standards that are in accordance with Brazilian Law 11.794/2008, which defined procedures to be employed in the scientific use of animals.

### Bacterial strains

The invasive *E*. *albertii* 1551–2 strain (intimin subtype omicron) and its isogenic mutants obtained in previous studies by our group (Table 1) were statically cultured in Luria Bertani broth (LB) for 18 h at 37°C. Antibiotics were added to select resistant strains as indicated in Table 1. The mutant strain 1551–2Δ*tir* was constructed employing the one-step allelic exchange recombination method [35]. Primers containing a 40-bp region homologous to the 5' and 3' ends of the *tir* gene and a specific sequence for the zeocin (zeo) resistance-encoding gene (*tir*-zeo-F ATG CCT 1 ATT GGT AAT CTT GGT CAT AAT CCC AAT GTG AGT GGT CAT CGC TTG CAT TAG AAA GG and *tir*-zeo-R TTA AAC GAA ACG ATT GGA TCC CGG CAC TGG TGG GTT ATT CGA ATG ATG CAG AGA TGT AAG) were used to amplify the Zeo cassette [36]. Amplicons obtained in the PCR reaction were electroporated into competent wild type bacteria harboring the pKOBEG-Apra plasmid. The selection of recombinant bacteria were done on Zeo-containing LB agar plates (60 μg/mL), and the *tir* deletion in the isogenic mutant was confirmed by PCR. In addition, the loss of pKOBEG-Apra plasmid was confirmed by testing the mutant strain for apramycin susceptibility.

**Table 1:** Bacterial strains.

Strain	Characteristics	Origin
*E*. *albertii* 1551–2 Nal^R^	Wild type strain (wt)	[21]
*E*. *albertii* 1551-*2eae*::Kn^R^	*eae* mutant (*eae*)	[21]
*E*. *albertii* 1551–2Δ*tir* (Zeo^R^)	*tir* mutant (Δ*tir*)	This study
*E*. *albertii* 1551-2escN:Kn^R^	*escN* (T3SS) mutant (*escN*)	[36]
*E*. *albertii* 1551-2*escN*::Kn^R^(pEscN)	*escN* mutant strain complemented with pACY184 carrying *escN* gene of tEPEC E2348/69 (pEscN)	[36,37]
*E*. *albertii* 1551–2Δ*fimA* (Zeo^R^)	*fimA* mutant (Δ*fimA*)	[36]
*E*. *albertii* 1551–2Δ*fimA*(pFimA) (Zeo^R^Amp^R^)	*fimA* mutant strain complemented with plasmid pBAD Myc carrying *fimA* gene of aEPEC 1551–2 (pFimA)	[36]
tEPEC E2348/69	tEPEC prototype strain (E2348/69)	[38]
R6	Rat *E*. *coli* strain that is able to translocate from lumen to extra-intestinal organs (R6)	[39]
*E*. *coli* K12 HB101	Non-pathogenic laboratory strain (HB101)	[40]

Nal^R^: nalidixic acid resistant (20 μg/mL); Kn^R^: kanamycin resistant (50 μg/mL); Zeo^R^: Zeocin resistant (60 μg/mL); Amp^R^: ampicillin resistant (100 μg/mL).

### Cell culture conditions

Caco-2 cells (ATCC HTB-37, purchased from Banco de Células do Rio de Janeiro, Rio de Janeiro, Brazil) and NIH 3T3 cells (ATCC CRL 2795, kindly provided by Dr. Beatriz Castilho, UNIFESP) were grown in DMEM (Gibco Invitrogen, USA) supplemented with 10% fetal bovine serum (Gibco Invitrogen, USA), 1% non-essential amino acids (Gibco Invitrogen, USA) and 1% antibiotics (Pen Strep—Gibco Invitrogen, USA). Raji-B cells (ATCC CCL-86, kindly provided by Dr. Roger Chammas, Universidade de São Paulo, São Paulo, Brazil) were cultured in RPMI-1640 (Gibco Invitrogen, USA) supplemented with 10% fetal bovine serum, 200 mM glutamine (Gibco Invitrogen, USA) and 1% antibiotics. The cell lines were cultured at 37°C in an atmosphere of 5% CO_2_.

### Adhesion and Invasion assays

Quantitative assessment of bacterial association and invasion was performed as described previously [24,41]. Briefly, differentiated Caco-2 cells were infected with 10^7^ colony-forming units (CFU) of *E*. *albertii* strain 1551–2 and its isogenic mutants for 6 h. Thereafter, cell monolayers were washed three times with phosphate buffered saline (PBS). While one set of monolayer-containing wells was lysed in 1% Triton X-100 for 30 min at 37°C, another set was treated with 100 μg/mL of gentamicin (Sigma, USA) for one hour at 37°C, and then washed 5 times prior to lysis. Following cell lysis, bacteria were resuspended in PBS and quantified by plating serial dilutions onto MacConkey agar plates to obtain the total number of cell-associated bacteria and of intracellular bacteria. The invasion indexes were calculated as the percentage of the total number of cell-associated bacteria that were located in the intracellular compartment. Assays were carried out in triplicate, and the results from at least three independent experiments were expressed as the percentage of invasion (mean ± standard error).

### Animals

Female Wistar-EPM rats, ~3 months-old and weighting 200–250 g, were obtained from the Central Animal Facility of Universidade Federal de São Paulo (UNIFESP). After 14 days of environment adaptation, stool samples were collected for coproculture and *E*. *coli* recovered from each animal were screened for the presence of the *eae* gene, which encodes the adhesin intimin, by PCR (AE11 5’-CCCGGCACAAGCATAAGCTAA-3’ and AE12 5’-ATGACTCATGCCAGCCGCTCA-3’, generating a fragment of 917 bp [42]). This procedure was performed to avoid the use of experimental animals that were colonized by either *E*. *coli* or *Citrobacter rodentium*, a murine pathogen that also promotes AE lesion formation [43]. Prior to the assays, animals were fasted for 24 h with access to water.

### *In vivo* organ culture (IVOC) bacterial colonization assay

For removal of ileum fragments, rats were held under anesthesia (pre-atropinization, induction of inhalation anesthesia with isoflurane and maintenance with intramuscular injection of 0.1 mL/100 g body weight ketamine + xylazine (4:1 1). After antisepsis, rats were subjected to median laparotomy for the collection of intestinal fragments of ~ 0.5 cm^2^. Briefly, ileal segments were removed, sectioned longitudinally at its antimesenteric border and placed onto a sterile filter paper with its serous portion facing the filter. This procedure allowed the exposure of the entire apical surface of the mucosa to the bacterial inoculum. Fragments were kept in Dulbecco’s Modified Eagle Medium (DMEM—Gibco Invitrogen, USA) supplemented with 10% fetal bovine serum (Gibco Invitrogen, USA) [44]. Fragments were infected with 10^10^ CFU for 6 h of incubation (37°C, 5% CO_2_); fragments were then washed, macerated and suspended in PBS and plated in serial dilution onto MacConkey agar plates containing 20 μg/mL nalidixic acid [21] for quantification (calculation of the total number of mucosa-associated bacteria). Infected IVOC preparations were also fixed for electron microscopy analysis.

### *In vivo* bacterial translocation assay

Animals were maintained under anesthesia (intramuscular injection of 0.1 mL/100 g body weight of ketamine and xylazine (4:1) during the en*tir*e procedure. Additional half dose of anesthetic was administered when necessary. Bacterial translocation (BT) was induced by a midline incision, oroduodenal cannulation, injection of 10^10^ CFU/mL resuspended in 10 mL of saline through the catheter, and bacterial retention for a period of 2 h, within a portion between the duodenum and ileum, by means of ligatures [39]. The *E*. *coli* rat strain R6, which is devoid of the DEC virulence genes, such as the *eae* gene, was used as a BT-positive control strain [39], while the non-pathogenic *E*. *coli* strain HB101 was used as BT-negative control. Bacterial inoculation causes a transient dilation of the small bowel, which disappeared within a short period. Blood (1 mL), mesenteric lymph nodes (MLN), spleen and liver were then collected, weighed, macerated and suspended in PBS. Subsequently, bacterial colonies were enumerated after plating serial dilutions onto MacConkey agar plates containing 20 μg/mL nalidixic acid, to estimate the number of translocated bacteria. The results were expressed by mean log_10_ values of CFU/g tissue.

### M-like cell differentiation

M-like cells were obtained as previously described [45–47] with modifications. Briefly, Caco-2 cells (10^5^ cells/filter) were seeded on the upper chamber of a Millicell filter (3.0-μm pore diameter, Millipore, USA) and kept in DMEM as described above for 10 days at 37°C in an atmosphere of 5% CO_2_. The lower chamber was also filled with DMEM. During this incubation period, transmembrane electric resistance (TEER) was measured every two days using the Millicell^®^ ERS (Electrical Resistance System, Millipore), until it reached ~420 mΩ. Afterwards, Raji-B cells (10^6^ cells/mL) were seeded at the Millicell lower chamber and cultured in RPMI-1640, as described above, for 6 days. In parallel, in some filters, Caco-2 cells were kept in monoculture for an additional 6 days (non-differentiated cells). Since galectin-9 is expressed on M cells but not on Caco-2 cell surface [47], NIH 3T3 (positive control), Caco-2 (negative control) and M-like cells were fixed and incubated with galectin-9 (M-20):sc-19294 (Santa Cruz Biotechnology, INC), followed by Alexa Fluor 488 (donkey anti-goat IgG, Invitrogen) incubation. Green fluorescent cells indicated the presence of galectin-9. Alternatively, donkey anti-goat IgG-TR (Santa Cruz Biotechnology, INC), combined with phalloidin-FITC (Sigma, USA) and DAPI (Molecular Probes, USA) were used.

### *In vitro* bacterial translocation assay

Bacterial suspensions (10^7^ CFU) in DMEM (as described above, except for 1% antibiotics) were inoculated in the upper chamber of filters bearing either M-like/Caco-2 or Caco-2 cells only for 6 h. Filters were transferred to a well containing fresh medium (DMEM without antibiotics) every hour and the medium from the lower chamber was collected for bacterial quantification at 6 h [46]. In parallel, the transmembrane electric resistance (TEER) was measured. At the end of the infection period, monolayers were washed with PBS and fixed for microscopy.

### Scanning electron microscopy (SEM)

For SEM, preparations were fixed in 2% glutaraldehyde, post-fixed in 1% osmium tetroxide, and dehydrated through a graded ethanol series (50, 70, 90, and 100%). After dehydration, preparations were dried by the critical point method, mounted onto SEM stubs, coated with gold and examined under SEM (QUANTA 250—FEI Company, Netherlands) at 12.5 kV [48].

### Transmission electron microscopy (TEM)

Infected monolayers and ileum fragments were first fixed in 2% glutaraldehyde (EMS, USA) for at least 24 h at 4°C. After primary fixation, cells and fragments were washed 3 times with PBS (10 min) and subjected to secondary fixation with 1% osmium tetroxide (EMS, USA) in 0.1 M sodium cacodylate buffer for 30 min. After being washed three times with distilled water, preparations were dehydrated through a graded ethanol series (50%, 75%, 85%, 95% and 100%), and propylene oxide (100%). Preparations were then gradually embedded in Araldite, which was allowed to polymerize for 24–48 h at 60°C. Ultrathin sections were placed on Formvar (EMS, USA) coated 200 mesh copper grids and stained with 4% aqueous uranyl acetate (Merck, Germany) and Reynold's lead citrate (Merck, Germany). Grids were examined under TEM (LEO 906E– Zeiss, Germany) at 80 kV [48].

### Statistical analysis

Differences in bacterial adherence, invasion percentages and translocation or differences in TEER of infected M-like cells were assessed for significance by using an unpaired, two-tailed *t* test (GraphPad Prism 4.0).

## Results

### Intimin, Tir and T3SS are essential for invasion of human intestinal cells cultured *in vitro*

**Fig 1:**
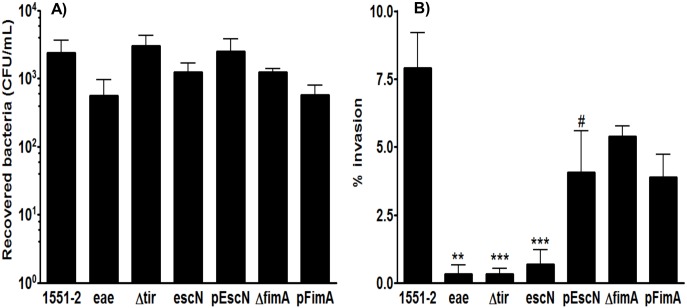
Quantitative assessment of association and invasion of *E*. *albertii* 1551–2 and its isogenic mutants to differentiated Caco-2 cells. Cells were infected with 10^7^ CFU for 6 h, monolayers were washed and one set of monolayer was lysed while another set was submitted to the gentamicin protection assay. The invasion indexes were calculated as the percentage of the total number of cell-associated bacteria that were located in the intracellular compartment. Assays were carried out in triplicate, and the results from at least three independent experiments were expressed as the percentage of invasion (mean ± standard error). A) Association. No statistical differences in the association of the wild type, mutant and complemented mutant strains were found. B) Invasion: (**) indicates statistical differences between 1551–2 and the intimin mutant (P = 0.0030). (***) indicates statistical differences between 1551–2 and Tir (P = 0.0002) or T3SS (P = 0.0004) mutants. (#) indicates statistical differences between T3SS mutant and complemented T3SS (P = 0.0238) strains.

Strain 1551–2 had been previously evaluated regarding its ability to invade differentiated Caco-2 cells [24]. In this study, Caco-2 cells were infected with bacterial suspensions of the wild type or its isogenic mutant strains (Table 1). Compared to the wild type strain the adherence index of mutant strains was not altered (Fig 1A) while the invasion index decreased significantly (Fig 1B), except for *fimA* mutation that did not affect the adherence or invasion indexes (Fig 1A–1B). These results confirm that *E*. *albertii* 1551–2 invasion depends on intimin and/or proteins injected by the T3SS, such as Tir, but not on T1P. Besides that, as the T3SS mutant did not inject Tir into Caco-2 cells, it is possible that, in the absence of its receptor, the 1551–2 intimin might recognize another host cell membrane structure as site for adhesion, but not for invasion, as confirmed by results obtained in invasion assays with 1551–2Δ*tir* strain. Complementation of T3SS mutant restored the invasion index to the wild type values (Fig 1B).

### *E*. *albertii* 1551–2 colonizes rat enterocytes in *in vitro* organ culture (IVOC)

To evaluate whether *E*. *albertii* 1551–2 could colonize the rat intestinal mucosa, ileal fragments (approx. 0.5 cm^2^) were individually infected with bacterial suspensions of the wild type or its isogenic mutant strains (Table 1). Methylene blue staining of the intestinal fragments was performed to confirm that all tissue layers were well preserved (S1 Fig).

**Fig 2:**
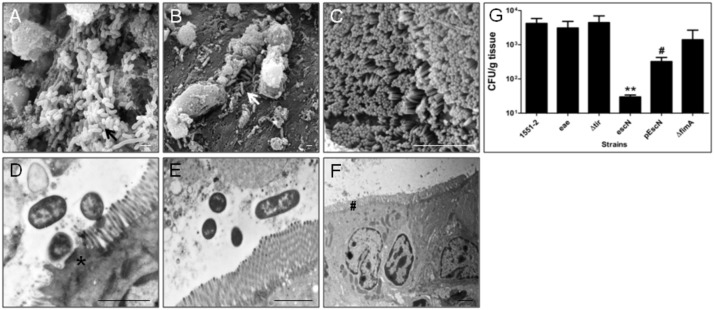
IVOC and *E*. *albertii* colonization assays. Ileal fragments collected from 3 Wistar-EPM rats were inoculated with 10^10^ CFU of each bacterial strain and incubated for 2 h at 37°C. SEM: (A) wild type *E*. *albertii* strain 1551–2 (black arrow), (B) T3SS mutant (white arrow), (C) non-infected control. TEM: (D) AE lesion underneath the wild type strain adherence site (#), (E) absence of AE lesion under the T3SS mutant interaction and (F) Non-infected control. (#) indicates preserved brush borders. Bars, 2 μm. (G) Quantification assay. After the incubation period, preparations were washed, macerated, and bacterial suspensions were diluted for seeding and CFU counting. (**) Significantly less adherent than wild type (p = 0.013), (#) significantly more adherent than T3SS mutant (P = 0.02). *eae*, intimin mutant; Δ*tir*, Tir mutant; *esc*N, T3SS mutant; pEscN, complemented T3SS mutant; Δ*fimA*, T1P mutant.

SEM images confirmed that the wild type strain strongly adhered to the intestinal mucosa (Fig 2A), whereas the T3SS-mutant comparatively showed a weaker adherence (Fig 2B). Non-infected fragments showed well-preserved bacterial-free brush borders (Fig 2C). Similarly to the wild type strain, the intimin, Tir and T1P mutants remained adherent to the intestinal mucosa (S2 Fig). Besides bacterial adherence, TEM images showed that the wild type strain caused AE lesions with characteristic pedestals underneath adhered bacteria on the rat mucosal surface (Fig 2D). In contrast, the T3SS-translocon mutant failed to cause AE lesions (Fig 2E), and non-infected fragments showed well-preserved bacterial-free brush borders (Fig 2F).

The number of CFU recovered from rat intestinal mucosa *in vitro* decreased significantly in the absence of the T3SS-translocon, while mutant strains deficient in intimin, Tir or T1P production, as well the T3SS mutant complemented strain, showed similar adherence levels in comparison with the wild type strain (Fig 2G).

### *E*. *albertii* strain translocates across rat intestinal barrier *in vivo*

**Fig 3:**
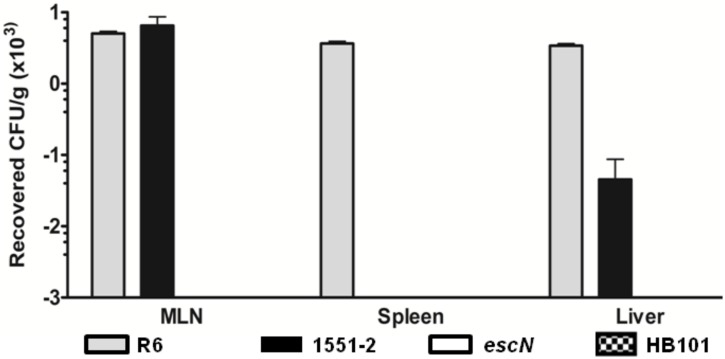
Bacterial translocation (BT) *in vivo*. *E*. *albertii* 1551–2 was recovered from the mesenteric lymph nodes (MLN) and liver. The T3SS mutant strain was not recovered from any tested organs. *E*. *coli* R6 (BT-positive control) was recovered from all examined organs while *E*. *coli* HB101 (BT-negative control) was not. *escN*, T3SS mutant.

To reduce the number of animals utilized in the next approach, we selected the T3SS-translocon mutant for *in vivo* comparison with wild type strain based on results obtained with the IVOC infection assay. Our results demonstrated that *E*. *albertii* 1551–2 reached the liver, while the T3SS-translocon mutant was not recovered from this organ. These findings suggest that, as a consequence of the reduced adhesion of this mutant to the intestinal mucosa, as observed *ex vivo*, fewer bacteria were available to cross the intestinal barrier, reach and survive in the MLN (Fig 3).

### *E*. *albertii* 1551–2 translocates across M-like cells

**Fig 4:**
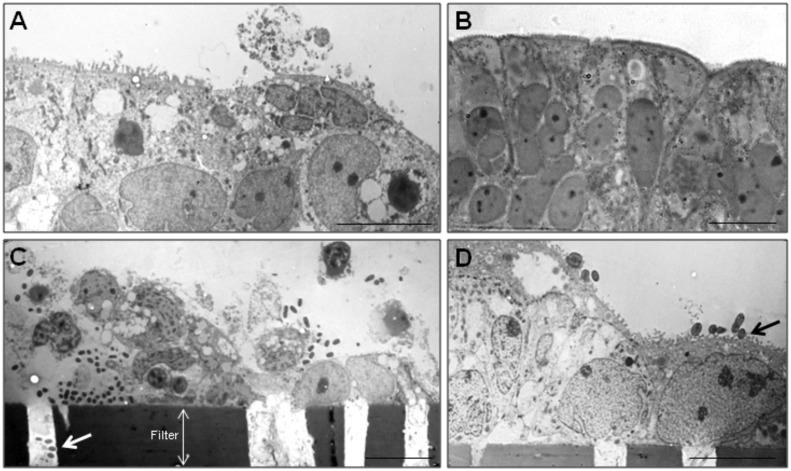
TEM of M-like and Caco-2 cells infected with *E*. *albertii*. M-like cells (A) showed few microvilli and a disorganized morphology, while on apical surface of differentiated Caco-2 cells (B) microvilli were still present. After *E*. *albertii* infection, M-like cells (C) allowed bacterial transcytosis (note bacteria crossing the filter membrane pore—white arrow), while in Caco-2 cells (D), *E*. *albertii* remained on the cells surface (black arrow). Bars, 10 μm.

Considering our results in the BT assay described in Materials and Methods and that pathogens such as *Shigella* species use M cells to cross the intestinal barrier, we performed *E*. *albertii* infection of M-like cells *in vitro* to identify the potential BT route employed *in vivo*. Prior to infection, we confirmed the conversion of part of the Caco-2 cells to M-like cells as described elsewhere [47], by demonstrating the expression of galectin-9 on M-like cell surface but not on Caco-2 cells (S3 Fig). Moreover, cellular morphology alterations [45] were observed on M-like cells, such as a reduced number of microvilli, flattened apical surface and disorganized cytoplasm (Fig 4A), while fully differentiated Caco-2 cells displayed preserved brush borders (Fig 4B). The presence of M-like cells significantly increased bacterial translocation (Fig 4C) as compared to differentiated Caco-2 cells (Fig 4D).

**Fig 5:**
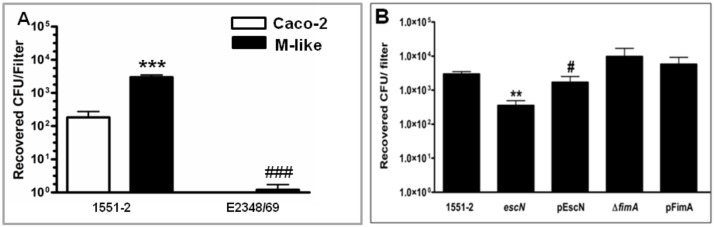
Bacterial Translocation through the M-like cells. Caco-2 cells were cultured for 10 days on Millicell filter. One group was co-cultured with Raji-B cell and control group were maintained in monoculture for 6 days. Then, upper chambers containing Caco-2 cells only or M-like cells were infected with *E*. *albertii* 1551–2, isogenic mutants or complemented strains for 6 h, or with tEPEC E2348/69 for 3 h. Media from the lower chamber were collected in three different periods during the incubation and seeded onto MacConkey agar for CFU counting. (A) Translocation of *E*. *albertii* and tEPEC through the Caco-2 and M-like cells: (***) indicates statistical differences between the two lineages infected with *E*. *albertii* strain (P<0.05). (##) indicates differences between M-like cells infected with 1551–2 and E2348/69 strains (P<0.05). (B) Translocation of *E*. *albertii*, isogenic mutants and complemented strains: (**) indicates differences between M-like cells infected with wt and T3SS mutant (P<0.05). (#) indicates difference between M-like cells infected with the T3SS mutant and its complemented strain (P<0.05). No statistical differences were found between in the transcytosis of wt and T1P mutant or between T1P mutant and its complement strains. *eae*, intimin mutant; *tir*, Tir mutant; *escN*, T3SS mutant; pEscN, complemented T3SS mutant; Δ*fimA*, T1P mutant. pFimA, complemented T1P mutant.

For quantitative *E*. *albertii* 1551–2 translocation assessment, tEPEC prototype strain E2348/69 was used as control [46]. We demonstrated that *E*. *albertii* translocation through M-like cells was significantly more effective than through differentiated Caco-2 cells (2,962.0±546.0 and 184.2±91.6, p = 0.0024, respectively) (Fig 5A), and as previously demonstrated [46], the presence of M-like cells did not increase the transcytosis of tEPEC E2348/69 in a significant manner as compared to differentiated Caco-2 cells (1.203±0.528 and 0.417±0.247, p = 0.1480, respectively). Additionally, *E*. *albertii* 1551–2 translocated through M-like cells more effectively than tEPEC E2348/69 (p = 0.033, Fig 5A). In order to exclude bacterial paracellular migration due to increased permeability as an invasion route, transepithelial electrical resistance was measured hourly during the infection period (S3 Fig). Comparison between M-like cells infected with the wild type or the T3SS mutant strains demonstrated a significant decrease in bacterial recovery (p = 0.0029, Fig 5B) with the latter strain, while complementation of the mutant strain restored its translocation capacity (p = 0.0418, Fig 5B and S4 Fig). Contrarily, non-significant differences between CFU recovered from T1P mutant and its complemented strains were observed with M-like cells (Fig 5B).

## Discussion

Previous data from our laboratory showed that the 1551–2 strain invaded HeLa cells [21] with invasion being dependent on the intimin-Tir interaction, since the intimin mutant (1551-2*eae*::Kn) was non-invasive [21]. Later on, we demonstrated that, in contrast with the wild type 1551–2 strain that displayed a localized pattern of adherence (formation of compact bacterial clusters) in HeLa cells, its T3SS-mutant adhered weakly, while the intimin mutant adhered, showing a T3SS-dependent diffuse pattern of adherence [36]. In addition, Pacheco et al., 2014 [24] showed that the 1551–2 strain invades, persists and multiplies inside differentiated Caco-2 cells up to 48 h.

In this work, we demonstrated for the first time that intimin, Tir and T3SS are essential for invasion of enterocytes *in vitro*, since mutations in the corresponding genes abolished bacterial uptake. Bacterial adherence was preserved in mutants, including the T3SS mutant, which did not adhere on HeLa cells in a previous study [36]. This fact might be due to the interaction between either intimin or T1P and Caco-2 cell surface receptors. It has been previously demonstrated that Tir and Map, and EspF can induce tEPEC invasion of HeLa and Caco-2 cells, respectively [49,50].

We have previously shown that an aEPEC strain, 1711–4, is able to translocate across the rat gastrointestinal barrier and be isolated from the MLN, spleen and liver [51]. The mechanisms promoting this bacterial translocation, however, are unknown. Generally, studies on colonization and infection by enteropathogens are conducted with Caco-2 cells, but although this cell line mimicries enterocytes from the human small intestine, it does not represent the complex intestinal mucosa, since it is devoid of the mucosal layer and other intestinal cell types. It was demonstrated that EHEC [52] as well as tEPEC E2348/69 [44] colonize human IVOC. More recently, Etienne-Mesmin et al., [53] demonstrated that EHEC colonize and translocate into ileum fragments from mice, where Peyer’s patches are available, but quantification was not performed. In the present study, we evaluated *E*. *albertii* capacity to colonize the rat intestinal mucosa in the IVOC model, to mimicry the first steps that lead to bacterial translocation from the intestinal lumen to the extra-intestinal sites demonstrated *in vivo*. We showed for the first time the interaction of *E*. *albertii* with rat intestinal mucosa *ex vivo*, which could be an alternative model to study AE-producing pathogens’ interaction with more complex intestinal tissues. In this model, colonization was detected after 30 min of infection, and invasiveness was revealed after 2 h, when *E*. *albertii* 1551–2 could be found inside the enterocytes. Additionally, we demonstrated that *E*. *albertii* adherence to the rat IVOC depends on T3SS, as previously demonstrated in human IVOC for tEPEC E2348/69 [44], but not on intimin, Tir or T1P, since in the absence of these genes, bacterial adherence was qualitatively and quantitatively preserved. Thus, the use of this model may optimize the selection of potentially invasive strains to be tested *in vivo*, thus reducing the number of animals used to assess the fate of invasive *E*. *albertii* from the intestinal lumen to extra-intestinal sites.

We selected the T3SS mutant to compare to the wild type strain, since this mutant strain had previously shown a significantly reduced capacity to interact with the host epithelium in an *ex vivo* model, losing the capacity to invade cultured intestinal cells *in vitro*.

It has been reported that some *E*. *albertii* strains isolated from birds are able to adhere and to invade HEp-2 cells [54] and to reach the liver and spleen of one day-old chicks *in vivo*, possibly by disrupting the intestinal barrier, despite the minor intestinal mucosa alterations [54]. In this study, using an *in vivo* bacterial translocation assay in rats, we recovered the *E*. *albertii* 1551–2 strain in the MLN and liver but not spleen, while the T3SS mutant completely lost translocation capacity. It has been reported that T3SS-dependent effectors such as EspF, Map and NleA disrupt tight junctions that contribute to the integrity of the intestinal barrier [55–57]. In addition, some infectious processes can disturb the intestinal epithelium, for example, neutrophil migration during inflammation; this event promotes a transitory epithelial barrier destabilization, which exposes the basolateral side, either allowing enterocyte invasion [58] or offering an alternative route for bacterial translocation from the intestinal lumen to extra-intestinal niches.

Based on our finding that *E*. *albertii* 1551–2 can reach the MLN and liver *in vivo* and that the invasion level through the basolateral surface is higher than at the apical surface of T84 cell monolayers [22], we investigated how *E*. *albertii* might cross the intestinal epithelium. It is well known that enteropathogens can reach basolateral receptors and promote enterocyte invasion *in vivo* by transcytosis through M cells [25,59]. According to Hase and coworkers [28], bacterial translocation depends on T1P-GP2 interaction, since isogenic mutant or non-T1P producer strains were unable to translocate through M-like cells. On the other hand, Inman and Cantey [60] described that a rabbit EPEC strain (RDEC-1) produced AE lesion on the M cell membrane, suggesting that AE lesions could prevent bacterial internalization, thus preventing transcytosis and antigen presentation, thereby delaying the immune response.

In this study, *E*. *albertii* 1551–2 translocation was significantly more effective through M-like cells than Caco-2 cells only. This could not be observed with tEPEC as previously demonstrated by [46]. We also demonstrated that translocation depended on functional T3SS, and that T1P mutation did not compromise bacterial translocation, contrary to what was found by Hase et al., [28]. These differences could be due to allelic FimH alterations in T1P in different strains. Therefore, these data suggest that *E*. *albertii* 1551–2 may reach the enterocyte basolateral surface *in vivo* after M cell translocation. Etienne-Mesmin et al., [53] also found that EHEC O157:H7 and O113:H2 and their respective intimin and Shiga toxin mutants translocated more effectively through M-like cells in comparison with Caco-2 cells. Cieza et al., [61] reported that the translocation of adherent-invasive *E*. *coli* (AIEC) through M-like cells depends on IbeA (an invasin); however, *E*. *albertii* strain 1551–2 is devoid of the *ibeA* gene (not shown) and other invasion-related genes [62], reinforcing that the bacterial translocation ability of this *E*. *albertii* strain is due to intimin-*Tir* interaction.

Altogether, our results demonstrated for the first time that both *ex vivo* and *in vivo* bacterial infection of rat intestinal mucosa are useful models to study *E*. *albertii* interaction with the host. We also showed that *E*. *albertii* 1551–2 may also cross the intestinal mucosa *in vivo* possibly using M cells as a route to reach extra-intestinal organs.

## Supporting Information

S1 Fig Light microscopy of rat intestinal mucosa prior to *ex vivo* infection. Ileal fragments (approx. 0.5 cm^2^) were stained with Methylene blue to confirm, by the presence of neurons from the myenteric plexus (Black arrows), that all tissue layers (as indicated) are well preserved. Magnification 400x.
(TIF)

S2 Fig IVOC and *E*. *albertii* isogenic mutants colonization assays. Ileal fragments collected from 3 Wistar-EPM rats were inoculated with 10^10^ CFU of each bacterial strain and incubated for 2 h at 37°C. SEM: (A) intimin mutant, (B) Tir mutant (white arrow), (C) Type 1 pilus mutant. Bars: A and B = 5 μm; C = 200 mm.
(TIF)

S3 Fig Caco-2 cells conversion into M-like cells and measurement of Transepithelial Electrical Resistance during *E*. *albertii* infection. To verify the M-like cell conversion, immunofluorescence staining was performed to detect Galectin-9 (green): A) NIH 3T3 cells (positive control), B) Differentiated Caco-2 cells, C) M-like cells. D-E, a monolayer containing M-like cells infected with *E*. *albertii* 1551–2 strain immunostained for Galectin-9 (red), combined with phalloidin-FITC and DAPI, for actin (green) and nucleic acid (blue), respectively. D) Merge image of actin and nucleic acid: insert (red square) indicates zoom of the white square area, showing actin accumulation underneath bacterial adhesion sites, E) Merge image of Galectin-9 and nucleic acid: insert (red square) indicates zoom of the white square area, showing bacterial adhesion site (Original Magnification: A-C 1,000x and D-E 400x). F) Transepithelial Electrical Resistance of monolayer containing M-like cells during infection with the 1551–2 strain. Non-significant differences were observed (p>0.05).
(TIF)

S4 Fig TEM of M-like and Caco-2 cells infected with T3SS mutant and complemented *E*. *albertii* strain 1551–2. After infection of M-like (A) and Caco-2 (B) cells with mutant strain, none or rare bacteria were observed adhered to the host cell surface. Complemented strain infecting M-like cells (C) presented intimate adherence and pedestal formation (AE lesion—arrow), while the same strain infecting Caco-2 cells (D) loosely adhered to the tips of cells microvilli. Bars, 1 μm.
(TIF)
