# Supplementary figures and images for: Escherichia albertii, a novel human enteropathogen, colonizes rat enterocytes and translocates to extra-intestinal sites
Source: PLoS One. 2017 Feb 8;12(2):e0171385. doi: 10.1371/journal.pone.0171385 (PMC5298312; doi:10.1371/journal.pone.0171385)

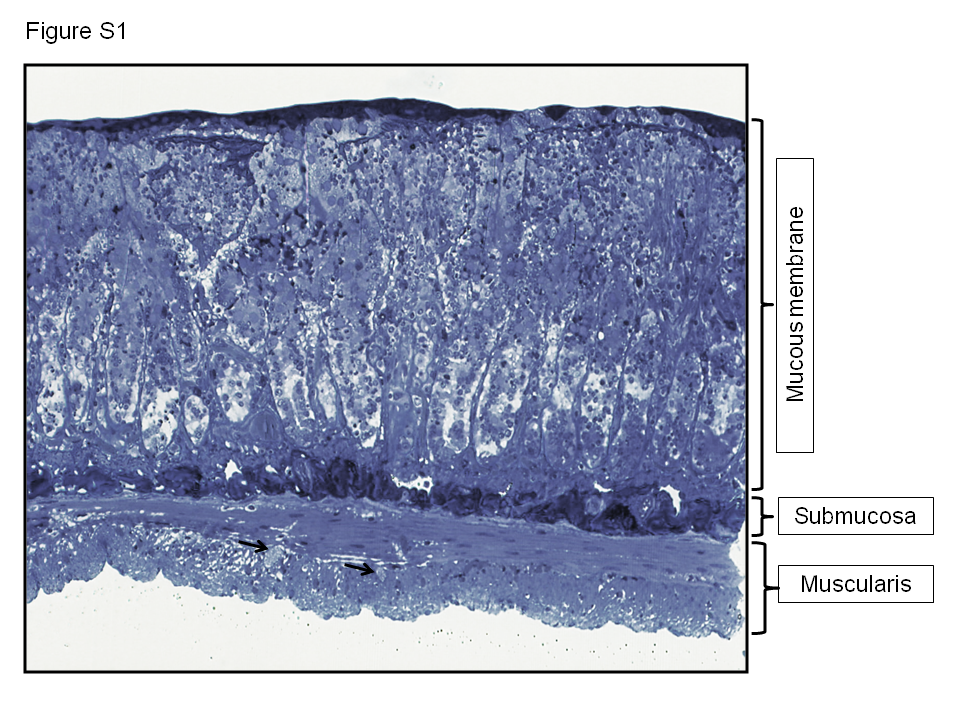

Supplement: S1 Fig — Ileal fragments (approx. 0.5 cm2) were stained with Methylene blue to confirm, by the presence of neurons from the myenteric plexus (Black arrows), that all tissue layers (as indicated) are well preserved. Magnification 400x. (TIF) [file pone.0171385.s001.tif]

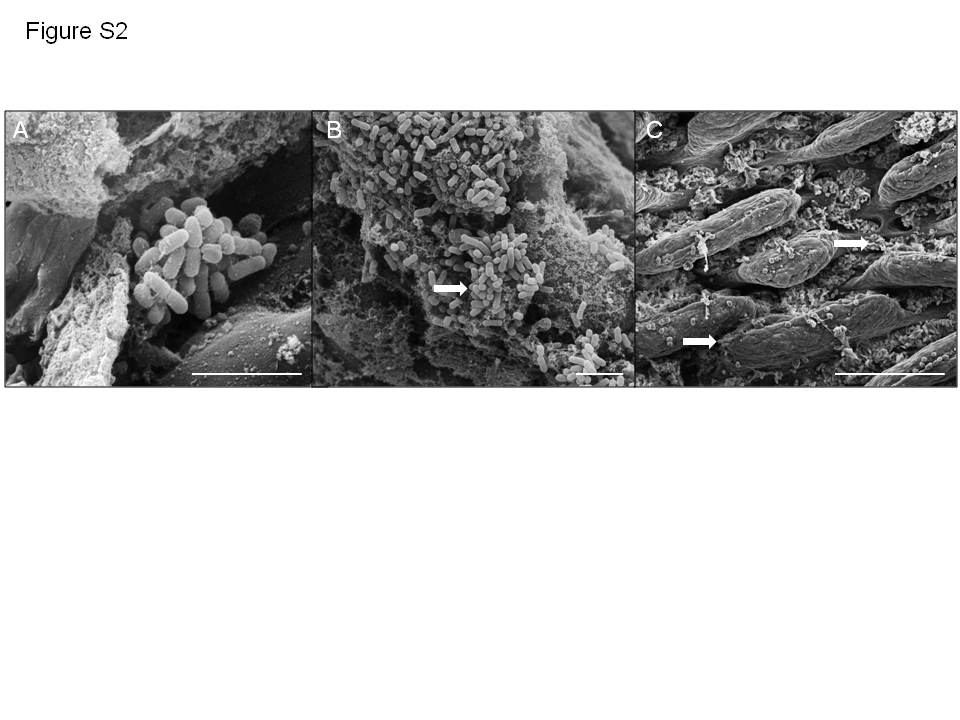

Supplement: S2 Fig — Ileal fragments collected from 3 Wistar-EPM rats were inoculated with 1010 CFU of each bacterial strain and incubated for 2 h at 37°C. SEM: (A) intimin mutant, (B) Tir mutant (white arrow), (C) Type 1 pilus mutant. Bars: A and B = 5 μm; C = 200 mm. (TIF) [file pone.0171385.s002.tif]

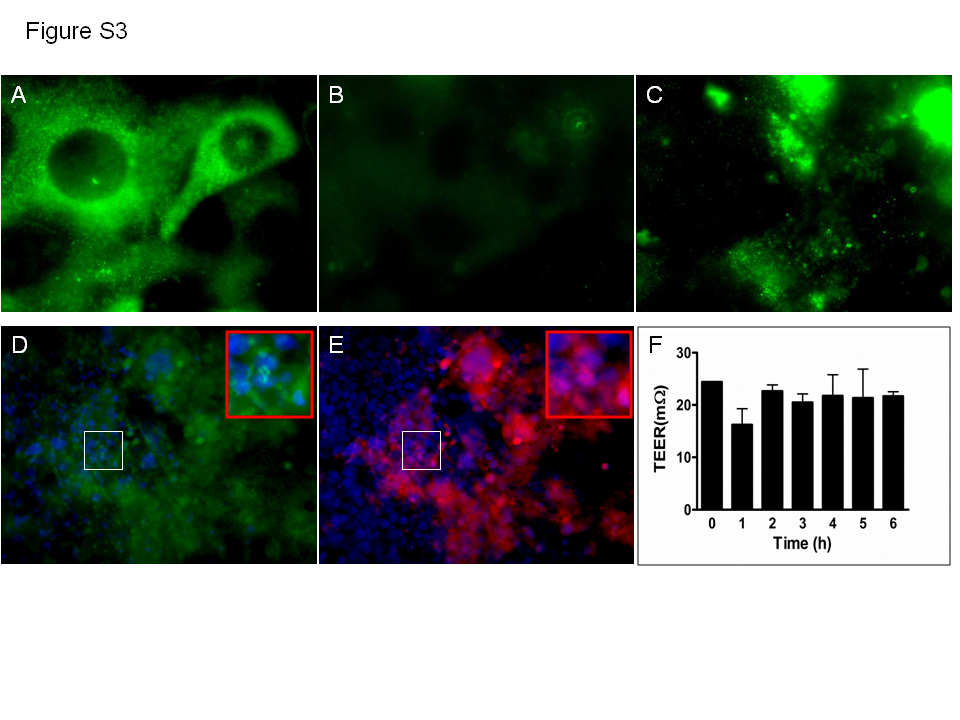

Supplement: S3 Fig — To verify the M-like cell conversion, immunofluorescence staining was performed to detect Galectin-9 (green): A) NIH 3T3 cells (positive control), B) Differentiated Caco-2 cells, C) M-like cells. D-E, a monolayer containing M-like cells infected with E. albertii 1551–2 strain immunostained for Galectin-9 (red), combined with phalloidin-FITC and DAPI, for actin (green) and nucleic acid (blue), respectively. D) Merge image of actin and nucleic acid: insert (red square) indicates zoom of the white square area, showing actin accumulation underneath bacterial adhesion sites, E) Merge image of Galectin-9 and nucleic acid: insert (red square) indicates zoom of the white square area, showing bacterial adhesion site (Original Magnification: A-C 1,000x and D-E 400x). F) Transepithelial Electrical Resistance of monolayer containing M-like cells during infection with the 1551–2 strain. Non-significant differences were observed (p>0.05). (TIF) [file pone.0171385.s003.tif]

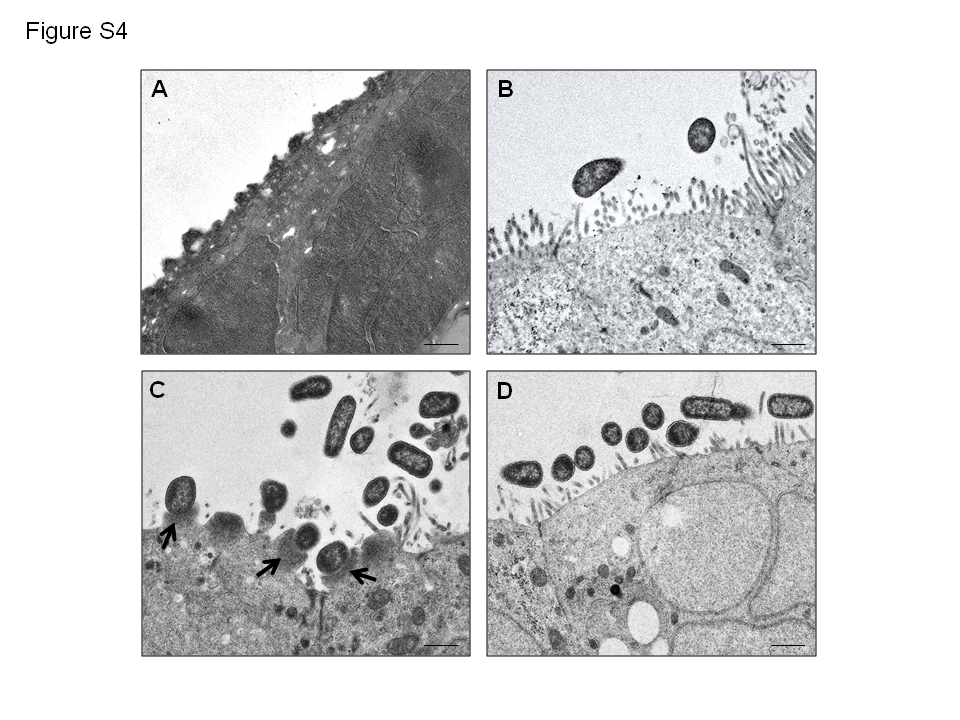

Supplement: S4 Fig — After infection of M-like (A) and Caco-2 (B) cells with mutant strain, none or rare bacteria were observed adhered to the host cell surface. Complemented strain infecting M-like cells (C) presented intimate adherence and pedestal formation (AE lesion—arrow), while the same strain infecting Caco-2 cells (D) loosely adhered to the tips of cells microvilli. Bars, 1 μm. (TIF) [file pone.0171385.s004.tif]
